# Weekend Effect and Mortality Outcomes in Aortic Dissection: A Prospective Analysis

**DOI:** 10.2478/jccm-2024-0014

**Published:** 2024-04-30

**Authors:** Cosmin Marian Banceu, Marius Harpa, Klara Brinzaniuc, Nicolae Neagu, Dan Alexandru Szabo, Diana Mariana Banceu, Hussam Al Hussein, Daiana Cristutiu, Alexandra Puscas, Alexandru Stan, Marvin Oprean, Adrian Popentiu, Marius Neamtu Halic, Horatiu Suciu

**Affiliations:** Doctoral School, George Emil Palade University of Medicine, Pharmacy, Science, and Technology of Targu Mures, Romania; George Emil Palade University of Medicine, Pharmacy, Science, and Technology of Targu Mures, Romania; Emergency Institute for Cardiovascular Diseases and Transplantation Targu Mures, Romania; Targu Mures Institute for Cardiovascular Diseases and Heart Transplantation, Targu Mures, Romania; Amherst College, Amherst, Massachusetts, USA; Faculty of Medicine, Lucian Blaga University of Sibiu, Sibiu, Romania; Swiss Federal Institute of Forest, Snow and Landscape Research WSL, Birmensdorf, Switzerland; Institute of Environmental Engineering, ETH Zurich, Switzerland; Swiss Federal Institute for Environmental Science and Technology - Eawag, Switzerland

**Keywords:** aortic dissection, weekend effect, risk factors, mortality outcome

## Abstract

**Background:**

Aortic dissection (AD) is a critical heart condition with potentially severe outcomes. Our study aimed to investigate the existence of a “weekend effect” in AD by examining the correlation between patient outcomes and whether their treatment occurred on weekdays versus weekends.

**Methods:**

Specifically, we prospectively analysed the effect of weekday and weekend treatment on acute AD patient outcomes, both before surgical intervention and during hospitalization, for 124 patients treated from 2019–2021, as well as during 6 months of follow-up.

**Results:**

The mean age of the study population was 62.5 years, and patient age exhibited a high degree of variability. We recorded a mortality rate before surgery of 8.65% for the weekend group and 15% for the weekday group, but this difference was not statistically significant. During hospitalization, mortality was 50% in the weekend group and 25% in the weekday group, but this difference was not statistically significant. Discharge mortality was 9.61% in the weekend group and 5% in the weekday group.

**Conclusions:**

Our findings suggest that there was no significant difference in mortality rates between patients admitted to the hospital on weekends versus weekdays. Therefore, the period of the week when a patient presents to the hospital with AD appears not to affect their mortality.

## Introduction

Despite the significant progress in medical care over recent decades, typical high rates of death related to aortic dissection (AD) continue to be a crucial issue [1, 2]. Although intraoperative mortality has decreased, postoperative mortality remains high due to comorbidities that can influence the patient's outcomes [1, 3, 4]

Since treatment is usually provided to the affected segment of the aorta only, leaving the rest of the aorta at risk, it is essential to have a deep understanding of the disease's etiology [5, 6]. With healthcare providers’ increased experience with aortic disease prevention, diagnosis, and treatment protocols, mortality rates have significantly decreased [7, 8, 9]. However, to provide surgical treatment and protect the patient from experiencing additional complications, an early diagnosis is necessary [10, 11]. Although there is an improvement in the survival rate, there are still persistently high mortality rates with unclear evidence. To improve overall patient outcomes in this medical conditions, recognition and management of these contributing factors might be crucial [12, 13].

Recent studies have shown that one of the key factors that influences overall patient survival is whether the patient's admission day is during the weekend or on a weekday [14, 15, 16]. Notwithstanding the availability of the healthcare system during the weekends, patients who receive treatments on weekends have higher morbidity and mortality rates [17, 18]. This might be a consequence of a more limited diagnostic investigation on weekends compared to weekdays. As a consequence, it is more difficult both to identify the underlying medical conditions of patients and to successfully implement therapeutic preventive measures meant to decrease immediate mortality.

Whether a patient is admitted to the hospital during the week or the weekend, the timing of their presentation is an important factor that should not be ignored and deserves further investigation. The absence of all medical staff from the unit throughout the weekend and the presence of only on-call medical staff can delay treatment of patients suffering thoracic aorta injury, indicating the need for this study. The various levels of experience of healthcare professionals should also be considered, as they may have a negative impact on treatment outcomes [19]. Before surgery, it is essential to identify potential risk factors and adopt initiatives to improve patient outcomes. It is difficult to overestimate how crucial this strategy is, and it deserves ongoing attention [20].

Regardless of the day of hospital admission, maintaining stability in AD is vital for effective treatment. The purpose of this research was to compare the outcomes of patients treated on weekdays and weekends to determine whether there was a difference in mortality before, during, and after invasive treatment for AD. A prospective analysis was performed with patients who received surgical treatment for AD in a single cardiac surgery department.

## Materials and Methods

This study aimed to assess the relationship between the day of the week and mortality in patients undergoing invasive treatment for AD. A prospective analysis was performed to determine the mortality rates before, during, and after treatment and to compare them between weekdays and weekends.

To assess whether the admission timing may affect the outcome of AD, the study included 124 patients surgically treated in a single tertiary cardiac surgery center from 2019–2021, with a 6-month follow-up.

Complete medical records gathered throughout the preoperative, perioperative, and postoperative phases of surgery are included in the data collected for this study. In total, these provide a comprehensive assessment of patients’ health state and outcomes throughout the surgical procedure. They contain detailed medical tests, laboratory findings, as well as information collected from various imaging techniques.

The inclusion criteria for this study were adult patients (over the age of 18) presenting with AD who gave their informed consent to participate in the study. The patients were treated on a 7-day/week basis. One distinguishing feature of the subgroup of patients in our study who arrived at the hospital in cardiac arrest was that they did not have a history of coronary artery disease. Rather, it was determined that an acute aortic dissection exacerbated by coronary malperfusion was the cause of the cardiac arrest. Crucially, cases of cardiac arrest with this etiology were purposefully included in our research, enabling a thorough analysis of the results and advancing knowledge of the intricacies involved with acute aortic dissection and how it affects coronary perfusion in the setting of cardiac arrest.

The exclusion criteria were patients who did not provide consent; were under the age of 18; were admitted to the hospital with associated medical comorbidities, such as chronic congestive heart failure or extracorporeal membrane oxygenation use preoperatively; and who required treatment for chronic heart diseases on either weekdays or weekends. Distinguishing between acute and chronic heart failure, with its distinct etiology, clinical manifestation, and prognostic consequences, was a crucial factor considered when defining the parameters of our research. Notably, we purposefully excluded patients with chronic heart failure from our analysis due to the recognized link between chronic heart failure and an increased mortality risk in the setting of several cardiac surgical procedures. The purpose of our study is to investigate the outcomes and factors influencing a specific patient population by concentrating on excluding patients who have chronic heart failure. These exclusion standards were justified by the necessity to preserve a homogeneous study group and guarantee that the results are reliable and relevant to the target groups.

Within the framework of this research, weekdays are defined as Monday through Friday, which makes up the traditional workweek, while weekends are defined as Saturday and Sunday. This classification was used to make it easier to classify different events, interventions, or results chronologically within the time frame investigated.

Deaths of patients that occurred while they were receiving treatment during hospitalization were referred to as “in-hospital mortalities.” The term “discharge mortality” refers to the proportion of patients who died during the 6-month follow-up period.

All patient data were analyzed following ethical standards, intensive care unit (ICU) protocols, and standard surgical operating protocols, and the study was conducted following the Declaration of Helsinki and was approved annually by the Ethical Committee of University (resolution No. 239 7225/07.10.2019, No. 878/23.04.2020, and No. 1359/10.05.2021).

Statistical analysis was performed using the IBM SPSS Statistics program (Version 16.0, released 2015 for Windows, Armonk, NY: IBM Corp., USA). Descriptive statistics were presented as percentage (frequency) for categorical variables and were analyzed using χ^2^ or Fisher's exact test as needed and median ± standard deviation for continuous and parametric variables. Descriptive statistics were often reported as absolute and relative frequencies for categorical variables. The mean ± standard deviation (minimum–maximum) was reported for continuous data. Odds ratios (OR) were presented with corresponding 95% confidence intervals (CI) and P values; P < 0.05 was considered statistically significant. Kaplan-Meier survival curves were used to determine long-term survival. Patients with unknown mortality were censored. Log rank (Mantel–Cox), Breslow (generalized Wilcoxon), and Tarone–Ware tests were performed to determine if there were any differences in mortality rates between patients admitted to the hospital on weekends and weekdays. This amount was the minimum required measurements or surveys (Minitab 20 Statistical Software, 2020. State College, PA, USA: Minitab, Inc., www.minitab.com). The number 124 was chosen as the appropriate sample size, leading to a statistical power of 0.841765 (or 85%). It was determined that the statistical power (S.P.) required to identify a significant effect according to the appropriate confidence level was at least 0.8.

## Results

**Fig. 1.**
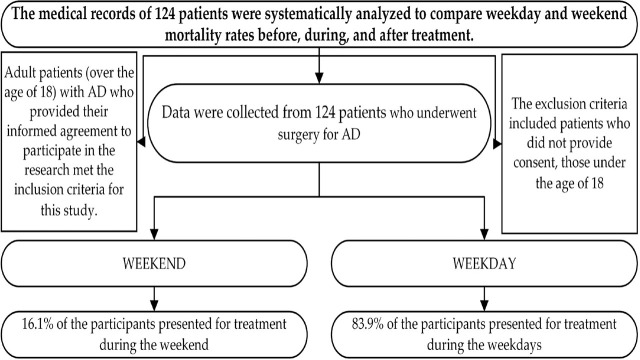
Flowchart of patients’ characteristics

Study participants included 124 patients who underwent surgery for AD (Figure 1).

The study provides an in-depth overview of the participants’ clinical and baseline characteristics, which can provide valuable insights into the factors that might have influenced their outcomes (Table 1).

**Table 1.** General characteristics measured before and after treatment (*N* = 124 patients)

		**Weekday**		**Weekend**		**Total**		**p-value**

		**Frequency (n)**	**Percent (%)**	**Frequency (n)**	**Percent (%)**	**Frequency (n)**	**Percent (%)**	
Presentation		104	83.87	20	16.13	124	100.00	< 0.01
Gender	Men	71	84.52	13	15.48	84	67.74	< 0.01
	Women	33	82.50	7	17.50	40	32.26	< 0.01

**Before Operation**								
Hemopericardium		34	85.00	6	15.00	40	32.26	0.09
Cardiac tamponade		3	72.22	5	27.78	18	14.51	< 0.01
Cardiogenic shock		11	84.61	2	15.38	13	10.48	0.07
Cardiac arrest		0	-	2	10.00	2	1.61	0.02
Mortality before surgery		9	75.00	3	25.00	12	9.68	< 0.01
Anticoagulation therapy		3	2.42	0	-	3	2.42	< 0.01
Antiplatelet therapy		17	89.47	2	10.53	19	15.32	0.07

**After Operation**								
Ascending aorta replacement		63	84.00	12	16.00	75	60.48	< 0.01
Hemiarch replacement		10	90.91	1	9.09	11	8.87	< 0.01
Aortic valve replacement		21	87.50	3	12.50	24	19.35	0.09
RCA reimplantation		7	77.78	2	22.22	9	7.26	< 0.01
LCA reimplantation		6	75.00	2	25.00	8	6.45	< 0.01
LAD bypass		4	100.00	0	-	4	3.23	0.06
RCA bypass		6	85.71	1	14.29	7	5.65	0.07
Bentall procedure		5	71.43	2	28.57	7	5.65	< 0.01

RCA, right coronary artery; LCA, left coronary artery; LAD, left anterior descending artery.

Particular conditions were found through preoperative analysis. Significant differences (p < 0.01) were found in the gender distribution and presentation frequency between weekdays and weekends. When we looked at preoperative variables, we found a variety of conditions in the study's groups. Hemopericardium was observed in 85% of cases managed during week-days (p=0.09), as well as cardiac tamponade in 72.22% of cases (p < 0.01) and cardiogenic shock in 84.61% of cases (p = 0.07). Before surgery mortality was linked within the weekday group at 75%, and in the weekend group at 25% (p < 0.01). Antiplatelet therapy was more frequently used in 89.47% (p = 0.07) of cases presented on weekdays, while anticoagulation therapy was obtained in 2.42% of cases. On analyzing postoperative interventions, we found that in 84% of cases operated on weekdays, ascending aorta replacement was performed, and in 16% of cases on weekends (p < 0.01). On weekdays, hemiarch replacement was carried out in 90.91% of cases, and on weekends, in 9.09% (p < 0.01). On weekdays 87.50% of cases and 12.50% on weekends had aortic valve replacement (p = 0.09). Reimplantation of the RCA was documented in 77.78% of week-day instances and 22.22% of weekend cases (p < 0.01). Seventy-five percent of weekday cases had LCA reimplantation, compared to 25% of weekend instances (p < 0.01). In all cases, LAD bypass was performed on week-days, while RCA bypass was performed in 85.71% of cases on weekdays and 14.29% on weekends (p = 0.07). Bentall operation was performed in 71.43% of patients during weekdays while on weekends in 28.57% of cases (p < 0.01).

Since there were not any statistically significant differences between the two groups concerning organ failure in the context of weekdays versus weekends, the laboratory tests were statistically analysed on the entire patient cohort (Table 2). The patients’ ages ranged widely, with a median of 62.5 years. The average left ventricular ejection fraction (LVEF) was 51.05% and also exhibited a considerable degree of variation. The mean estimated glomerular filtration rate (eGFR), which assesses renal function, was 71.30 mL/min/1.73 m^2^, which was within normal limits, but it varied widely. Liver function exhibited levels above the normal range and considerable variation. Hemoglobin levels were slightly below the normal range and did not vary substantially. White blood cell numbers were within the normal range and did not vary substantially, while platelet counts were normal but variable.

**Table 2.** Admission timing laboratory data of patients with Stanford A dissection

	**Mean**	**Minimum**	**Maximum**	**Std. deviation**
Age	62.53	17	86	12.866
LVEF (%)	51.05	0	60	10.434
Creatinine level (mg/dL)	1.25	0.59	6.69	0.789
eGFR (mL/min/1.73 m^2^)	71.30	7.9	216.6	23.418
GOT (U/L)	79.82	10.0	2638.0	289.399
GPT (U/L)	71.13	6.0	2067.0	240.204
Hemoglobin (g/dL)	12.50	5.4	26.1	2.601
Thrombocytes (103/μL)	224.92	20.7	996.0	122.128
Leukocytes (103/μL)	11.33	2.0	24.4	4.838

LVEF: left ventricular ejection fraction; eGFR: estimated glomerular filtration rate; GOT: glutamic oxaloacetic transaminase; GPT: glutamic pyruvic transaminase.

In Table 3, mortality events before surgical intervention are summarized. The weekend group had a lower rate of death than the weekday group. To evaluate the significance of this difference, three statistical tests were applied, but there were no statistical differences between the weekend and weekday groups.

**Table 3.** Mortality before surgery

	**Total present**	**No. of death events**	**Percent**	**p-value**		

				**Log rank (Mantel–Cox)**	**Breslow (generalized Wilcoxon)**	**Tarone–Ware**
Weekend	20	3	15.00	0.272	0.196	0.234
Weekday	104	9	8.65			

**Fig. 2.**
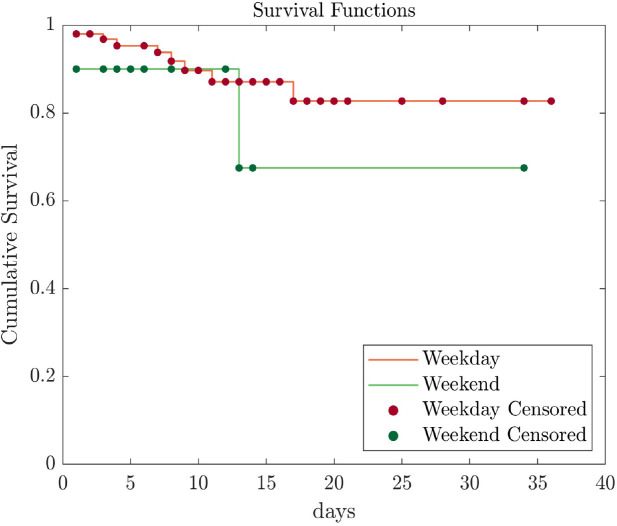
Kaplan–Meier representation of in-hospital mortality.

Kaplan–Meier survival analysis was used to compare in-hospital mortality rates between weekday and weekend patients (Figure 2).

Both patient groups presented a similar statistical pattern: comparable downward trends in their survival probabilities.

The results indicated that both patient groups presented a similar statistical pattern: comparable downward trends in their survival probability. The results of the analysis of in-hospital mortality data, including the numbers of patients, death events, and death event percentages, for both the weekend and weekday groups, are summarized in Table 4. The weekday group consisted of 104 patients, 52 death events, and a death event percentage of 50%. The weekday group comprised 20 patients, with 5 death events and a death event percentage of 25%. For patients who were admitted to the hospital on weekdays and died during their hospital stay, the mean time to death was 15.8 days (95% CI, 12.7–18.9). For patients who were admitted to the hospital on weekends and died during their hospital stay, the mean time to death was 18.1 days (95% CI, 7.7–28.6). Log-rank (Mantel–Cox), Breslow (generalized Wilcoxon), and Tarone–Ware tests were performed to determine if there were any differences in mortality rates between patients admitted to the hospital on weekends and weekdays. No statistically significant differences were found in in-hospital mortality between these two groups (Table 4).

**Table 4.** In-hospital mortality

	**Total present**	**No. of death events**	**Percent**	**Mean**				**p-value**		

				**Estimate**	**Std. error**	**95% confidence interval**		**Log rank (Mantel–Cox)**	**Breslow (generalized Wilcoxon)**	**Tarone–Ware**

						**Lower bound**	**Upper bound**			
Weekend	20	5	25.00	18.138	5.350	7.653	28.623	0.260	0.209	0.232
Weekday	104	52	50.00	15.806	1.599	12.671	18.941			

Data on immediate mortality were collected. In the weekday group, there were 104 patients, with 10 death events, resulting in a death event percentage of 9.61%. The weekday group comprised 20 patients, with 1 death event, resulting in a death event percentage of 5%. For patients who were admitted to hospital during the weekend, received surgical treatment, and died during the follow-up period, the mean time to death was 29.5 days (95% CI, 25.7–33.2). For the patient who was admitted to hospital on a weekday, received surgical treatment, and died during the follow-up period, the time to death was 34 days.

The analysis performed to determine if there were differences between weekend and weekday groups in mortality during the follow-up period showed no statistically significant differences (Table 5). In this case, no Kaplan–Meier survival analysis was performed given the reduced sample size, which did not permit consistent conclusions.

**Table 5.** Discharge mortality

	**Total present**	**No. of death events**	**Percent**	**Mean**				**p-value**		

				**Estimate**	**Std. error**	**95% confidence interval**		**Log rank (Mantel–Cox)**	**Breslow (generalized Wilcoxon)**	**Tarone–Ware**

						**Lower bound**	**Upper bound**			
Weekend	20	1	5.00	34.000	0.001	34.000	34.000	0.729	0.342	0.369
Weekday	104	10	9.61	29.456	1.912	25.710	33.203			

## Discussion

A patient's life is immediately affected by the catastrophic cardiovascular condition of AD. Understanding every factor that directly affects mortality rates is important since admission timing of a patient's to a hospital can have a significant impact on the course of treatment [21].

It is still necessary to identify other risk factors that might increase mortality rates besides the severity of aortic disease and its direct impact on treatment outcomes. Previous research has investigated the link between acute or chronic patients and arrival times at the medical facility, such as on weekends or weekdays, without general agreement [22, 23, 24].

In this study, the majority of patients were male and presented for treatment on weekdays. Patients with more complicated pathologies, such as hemopericardium, cardiac tamponade, cardiogenic shock, and cardiac arrest, were more likely to experience adverse outcomes. Additionally, the presence of anticoagulation or antiplatelet therapy added another layer of complexity to the patient's condition and may have contributed to the higher mortality rate observed in the study. The surgical interventions performed on patients involved in this study included ascending aorta replacement, hemiarch replacement, aortic valve replacement, RCA and LCA reimplantation, myocardial revascularization, and the Bentall procedure. The analysis indicated that the in-hospital mortality rate was higher than during the follow-up period. It is important to note the impact of the pathological complexity of these patients on both the surgical intervention and the outcome. Understanding these complexities is crucial for improving patient care and outcomes in cases of AD.

The associated medical condition could negatively affect patients’ outcomes, whether they receive elective or emergency aortic surgery, or whether they receive treatment on a weekday or a weekend [25].

Aortic dissection has been associated with a variety of etiological factors, such as male gender, age, genetics, hypertension, aortic valve diseases, and abnormalities of collagen tissue [26, 27, 28, 29]. Understanding the roles of these factors makes it possible to treat the condition, extend patient monitoring for those who are susceptible, develop targeted therapeutic approaches, and prepare patients for elective treatments when they meet the criteria for surgical indication.

Understanding a patient's health status and the need for additional intervention largely depends on the conclusions of the analysis. The results of this study indicated that the studied population's average age was high and that there was substantial variability in age. Multiple factors, including genetics, lifestyle, and overall health, could be responsible for the age range observed [30].

LVEF, which measures how efficiently the heart can pump blood, had a high standard deviation, which indicated a high level of variability. LVEF variation may be caused by a variety of conditions, such as aging, heart disease, and cardiovascular disease.

With a mean value and a low standard deviation, the creatinine levels in the study population showed low variability. This might indicate that the function measurements of the studied patients were comparable. The eGFR, by contrast, showed a high standard deviation, indicating a high level of variability, which may have been caused by several factors affecting kidney function. The liver function parameters showed high variability, which could have been a result of various factors affecting liver function. Similar levels of hemoglobin production among the study population were suggested by the low standard deviation of hemoglobin levels. The platelet counts, however, had a high standard deviation, indicating a high degree of variability, which may have been caused by a variety of factors that influence blood clotting. The white blood cell counts also had a low standard deviation, indicating that patients in the research population generated white blood cells at similar rates. The findings of our study provide information on the health of the group under observation and highlight the need for additional research to fully comprehend how these characteristics affect the prognosis of individuals with similar diseases.

Even if a genetic diagnosis does not exist, the pathogenesis may suggest different genetic syndromes, such as Marfan and Ehlers–Danlos syndromes, due to the disease's agelessness and premature development. Furthermore, this could be an indication of long-term conditions, such as atherosclerosis or uncontrolled hypertension, making it an important factor that should not be overlooked [31].

As AD has a high death rate—an estimated 50% during the first hour of onset—constant vigilance of the condition is essential [32]. This accentuates the urgency and importance of the condition since there is a high chance the patient will not arrive at the hospital in time for prompt and effective treatment. In particular, the occurrence of preoperative and postoperative events must be considered when analyzing mortality and its reduction in relation to inpatient and outpatient care.

To identify and manage risk factors and circumstances that could influence a hospitalized patient's mortality, such evaluation is required [33, 34, 35, 36]. Due to the high rate of death associated with this disease, the treatment approach in the case of an emergency presentation of a patient with an AD must be concentrated on the segments that are eligible for therapeutic intervention. Early recognition and treatment of known risk factors in these circumstances may make the difference between life and death [37, 38, 39, 40, 41, 42].

The results of the statistical analysis failed to show a significant difference between the mortality events before surgical intervention that occurred in the weekend group and the weekday group. The three tests used (log rank (Mantel–Cox), Breslow (generalized Wilcoxon), and Tarone–Ware) yielded *p*-values greater than 0.05, indicating a lack of statistical significance in the difference in death event percentage between the two groups. Although the percentage of death events was higher in the weekday group, the analysis did not indicate that this difference was statistically significant.

It is well documented that patients who arrive for elective treatment are managed better and have a higher chance of surviving than patients who seek emergency medical care. The affected aortic segment must always be identified to determine the best treatment strategy and establish any associated diseases and risk factors [43, 44].

The patient could develop hemodynamic instability while being transferred to the cardiac surgery department, which may increase overall mortality [45–46].

Our data analysis, which examined the association between in-hospital death rates and admission timing (weekend versus weekday), revealed no statistically significant difference in the mortality rates between the two groups. We also investigated the link between admission timing and immediate mortality (death within 6 months of release). There was no relationship between the admission timing and mortality rate, but further research is needed to validate these results and determine the underlying reasons for the correlation. This study provides valuable insights into in-hospital mortality patterns and has the potential to inform future research and medical practices aimed at improving knowledge of patient outcomes.

Based on the findings of the current study, there were no significant differences in in-hospital mortality rates among patients with AD who arrived on weekends and those who arrived on weekdays. This indicates that the causes of the death rates observed in these diseases are complex and multifactorial and that more research is required to identify and address the relevant factors. These findings shed light on the need for a comprehensive understanding of the risk factors, clinical procedures, and types of surgery related to AD to provide consistent and optimal patient outcomes each day of the week. Our observations, along with those from previous research, provide critical guidelines for additional studies to identify undiscovered risk factors that affect AD patients. These findings support ongoing efforts to improve patient outcomes as well as our knowledge of this complex disease, given that AD is a potentially fatal disorder that continues to inspire great interest and research in the medical community.

### Limitations

It is important to note that the study presented here was limited by the exclusion of patients who died before hospitalization, which may have impacted the overall results. Additionally, the study was conducted in a single cardiac surgery department, and the limited follow-up period of 6 months after surgery, as determined by the national registry, prevented long-term analysis.

## Conclusions

The results of the present study indicated that there was no significant difference in mortality between patients diagnosed with AD who presented on weekends versus weekdays. To further advance the field and enhance our understanding of these life-threatening diseases, it is imperative to continue investigating the factors that influence surgical outcomes. This objective can be achieved by conducting multicenter studies, which will broaden the scope and depth of analysis beyond the limitations of a single cardiac surgery center. In addition, the establishment of national registries and the implementation of preventative programs could contribute to reducing the mortality rate associated with AD. This study provides valuable insights into the mortality rate of AD and has the potential to inform future research and medical practices, shedding light on crucial areas for improving patient outcomes and shaping effective treatment strategies.
